# Vertigo in the Elderly: A Systematic Literature Review

**DOI:** 10.3390/jcm12062182

**Published:** 2023-03-11

**Authors:** Virginia Fancello, Stavros Hatzopoulos, Giuseppe Santopietro, Giuseppe Fancello, Silvia Palma, Piotr Henryk Skarżyński, Chiara Bianchini, Andrea Ciorba

**Affiliations:** 1 ENT & Audiology Unit, Department of Neurosciences, University Hospital of Ferrara, 44121 Ferrara, Italy; 2 Department of Otorhinolaryngology, Careggi University Hospital, 50134 Florence, Italy; 3 Department of Otorhinolaryngology, University Hospital of Modena, 41125 Modena, Italy; 4 Institute of Sensory Organs, 1 Mokra Street, 05-830 Kajetany, Poland; 5 Institute of Physiology and Pathology of Hearing, 10 Mochnackiego Street, 02-042 Warsaw, Poland; 6 Department of Heart Failure and Cardiac Rehabilitation, Medical University of Warsaw, 8 Kondratowicza Street, 03-242 Warsaw, Poland

**Keywords:** vertigo, elderly, etiology, vestibular disorders, BPPV, endolymphatic hydrops, falls

## Abstract

*Background:* Dizziness and vertigo are among the most prevalent complaints in the elderly and have a major negative influence on (i) the perception of the quality of life; and (ii) the risk of falling. Due to population aging, particularly in wealthy nations, vertigo represents a growing issue and a serious public health concern. In order to approach the patient correctly and to offer the best treatment options, it is mandatory to identify vertigo’s underlying causes. The aim of this paper was to identify the different etiologies of vertigo and possibly their frequency in the elderly population, by reviewing the scientific literature of the last decade (2012–2022). *Methods:* A systematic review was performed according to PRISMA guidelines, searching the Medline database from January 2012 through to December 2022. The search identified 1025 candidate papers, but after the application of specific selection criteria, only five were considered for further analysis. *Results:* A total of 2148 elderly patients (60–90 y old) presenting with vertigo were reported in the selected papers. A total of 3404 conditions were identified as the cause of vertiginous symptoms, (some patients presented multiple etiologies). All major diagnoses were categorized into different subgroups: the most common origin of vertigo was represented by audio-vestibular disorders (28.4%), followed by cardiovascular (20.4%) and neurological diseases (15.1%). Furthermore, 9.1% of patients were diagnosed with psychiatric conditions, whilst ophthalmologic and musculoskeletal disorders accounted for 7.5% and 6.3% of the cases respectively. Medication adverse effects and metabolic-related diseases were also considered among the causes. For 3.4% of cases the etiology remains unclear. Conclusions: Audio-vestibular disorders represent the most frequent cause of vertigo in the elderly. The etiologies affecting the vertigo patient must be defined in order to identify potential life-threatening conditions, such as cardiovascular and neurological disorders, which according to the data of this review constitute the second and third common causes of vertigo. A multidisciplinary strategy, involving different specialists (such as ENTs, Neurologists, Cardiologists, Geriatricians) is recommended for the correct assessment of these disorders.

## 1. Introduction

Vertigo represents one of the most common problems in the elderly; the term vertigo usually describes a sense of spinning and motion and differs from the term dizziness (floating sensation) and/or imbalance (loss of balance, unsteadiness), even if in the clinical practice all these terms are often used as synonyms. The presence of many scientific terms describing the clinical features of a dizzy patient confirms the many possible underlying causes. Among the multiple etiological causes of vertigo-episodes in the elderly, vestibular disorders are frequently reported as the most common. The age-related deterioration of vestibular function seems to be linked to a decreased number of vestibular hair cells and neurons, although alterations affecting the central pathways are also reported [1,2]. Despite this evidence, a cross-sectional analysis of the US National Health Care Survey, focusing on clinical pathways for balance disorders in older adults surprisingly revealed that an ENT/vestibular consultation has been performed in only 16.8% of elderly subjects with vertigo, even though audio-vestibular complications account for at least 21.5% of the cases [3]. Due to the presence of many possible vertigo etiologies, a systematic and structured approach is always valuable and crucial in the identification of the underlying pathologies and the choice of the best treatment and rehabilitation program. Moreover, very often the course of vertigo becomes chronic or recurrent and the subsequent psychological issues (fear about vertigo onset), comorbidities and concomitant symptoms, can affect the patient’s ability to manage these aspects in daily life [4].

Thus, with the constant aging of the population, especially in the developed countries, vertigo complications are becoming a public health issue, significantly impacting the quality of life and severely increasing the risk of falls, which are estimated to cause 50% of all unintentional deaths in patients of this age group [5].

The objective of this paper was to identify the different vertigo etiologies in the elderly population and possibly their relative frequency, by reviewing the scientific literature during the last decade.

## 2. Materials and Methods

A literature search of English-language studies on vertigo in elderly patients was performed using the Medline database. The time span of the search was set to 10 years i.e., 2012 to December 2022 and the aim was to select the most up-to-date studies, possibly, with similar diagnostic criteria.

The Mesh terms “Vertigo” and “Dizziness” were used in combination to additional filters such as publication year and the subject’s age. The query resulted in 1025 candidate papers on which the following criteria were applied:

Inclusion criteria:

- Original studies on cohorts >50 patients (in order to identify studies with adequate sample size; for additional details see the paper by Dworkin et al. [6]);
- Studies on elderly subjects, defined according to World Health Organization as those aged 60 years and above (https://www.who.int/health-topics/ageing#tab=tab_1, accessed on 30 December 2022);
- Studies including audio-vestibular diagnoses;

Exclusion criteria:

- Studies containing duplicated data from other published work;
- Cohort of patients <50;
- Studies published in a non-English language;
- Studies not including audio-vestibular diagnoses;
- Studies analysing only specific subgroups of diagnoses;
- Studies published before 2012.

From the initial candidates, only five papers met the inclusion criteria.

**Figure 1:**
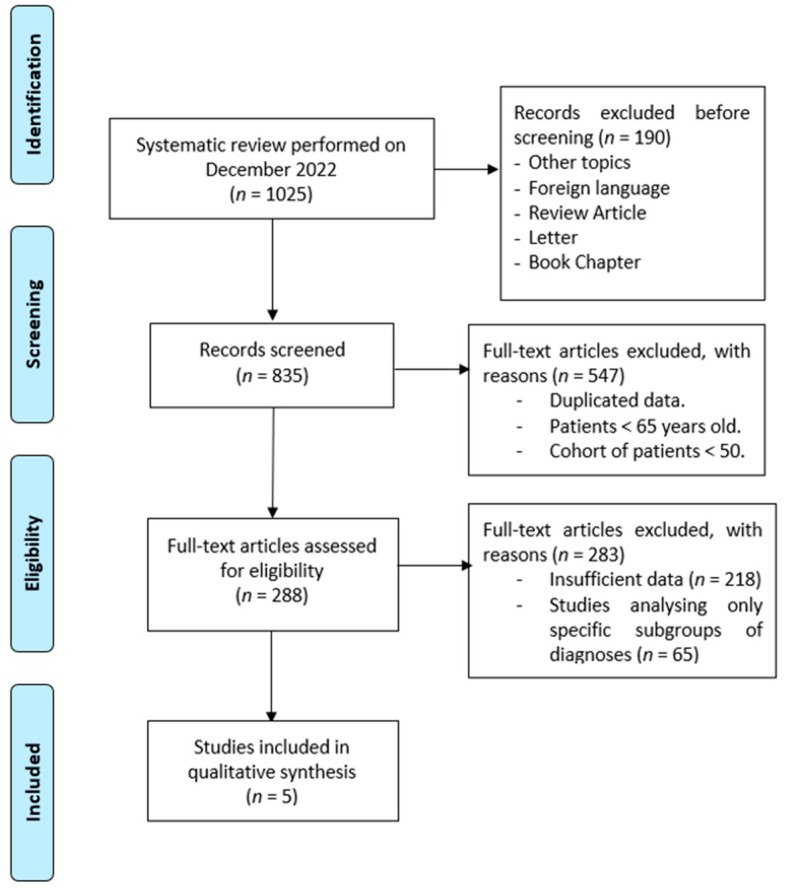
Literature evaluation and selection, according to PRISMA criteria (http://www.prisma-statement.org/).

The review was conducted using the Preferred Reporting Items for Systematic Reviews and Meta-Analyses (PRISMA) guidelines. The flow diagram is illustrated in Figure 1.

The review has been registered on Prospero with number 403542.

## 3. Results

The analysis of the five candidate papers considered data from 2148 elderly patients presenting vertigo. All the selected studies were prospective. The year of publication ranged from 2014 to late 2022. (see Table 1).

Overall, the total number of conditions identified by the selected studies, as the cause of vertigo, was 3404, because a number of patients presented multiple etiologies.

The patient’s age ranged from 60 to 90 y old and the male to female patient ratio was 1:1.6. The assessed subjects were derived from different continents and countries (Europe, South America, Asia); the setting of the studies is not homogeneous, ranging from a primary to a tertiary care centres or University Hospitals. Also, the approach to vertigo patients has been multidisciplinary (including assessments by Neurologists, ENTs, and Geriatricians).

**Figure 2:**
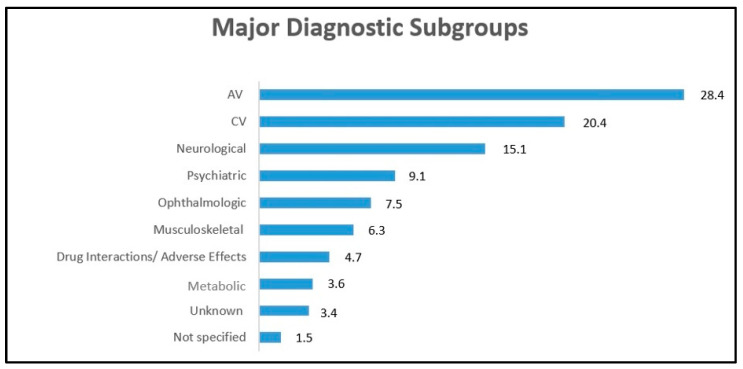
Different etiologies of vertigo. AV = audio-vestibular, CV = cardiovascular. Numbers are expressed in percentages.

All main diagnoses were categorized into different subgroups (see Figure 2): the most common cause of vertigo was represented by audio-vestibular disorders (28.4%), followed by cardiovascular (20.4%) and neurological diseases (15.1%).

**Figure 3:**
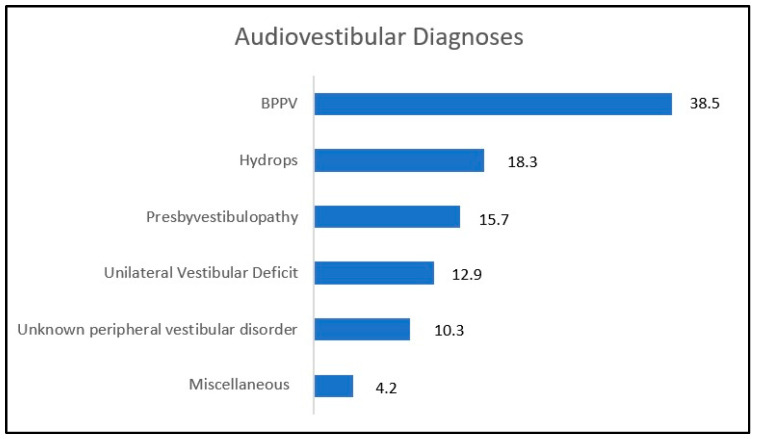
Audiovestibular diagnoses within the selected studies. All numbers are shown in percentages.

Audio-vestibular (AV) disorders were identified as the most frequent cause of vertigo. Further data analysis revealed how, among the AV disorders, benign paroxysmal positional vertigo (BPPV) is very common in older adults, representing the most frequent cause of vertigo across all patient groups (see Figure 3) [12]. Numerous studies have suggested that the incidence of BPPV increases with age, possibly due to the inner ear aging and particularly to otoconia degeneration; due to their reduced density, otoconia are more likely to be pathologically discharged from the utricular macula [13,14,15].

Not surprisingly, endolymphatic hydrops was the second most frequent AV disease, accounting for around 18%, considering the evolution of the pathology over time until the loss of vestibular function.

Presby-vestibulopathy [16], a chronic vestibular syndrome characterized by the presence of bilaterally reduced function of the vestibulo-ocular reflex (VOR), accounted for a considerable percentage (16%), alongside unilateral vestibular deficit (13%) and unspecified peripheral vestibular disorders (10%), as reported by Agrawal [2].

The miscellaneous category, shown in Figure 3**,** includes cases of vestibular paroxysmia, perilymphatic fistulae, cholesteatoma and toxic inner ear damage.

Cardiovascular (CV) disorders were only considered when reported among the primary reasons for vertigo.

**Figure 4:**
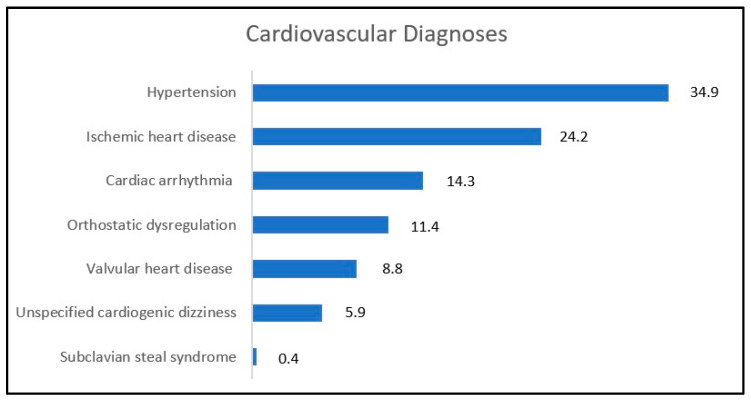
Cardiovascular diagnoses within the selected studies. All numbers are shown in percentages.

Blood pressure dysregulation (especially hypertension but also orthostatic dysregulation) has been identified as the main cause of the vertigo symptoms in 34.5% of CV diagnoses, followed by ischemic heart diseases, arrhythmias, and valvular heart diseases (see Figure 4).

However, since the prevalence of CV problems rises with age, they frequently represent a common comorbidity in the elderly, in particular considering that hypertension affects around 70% of persons above the age of 65, according to the literature [17].

**Figure 5:**
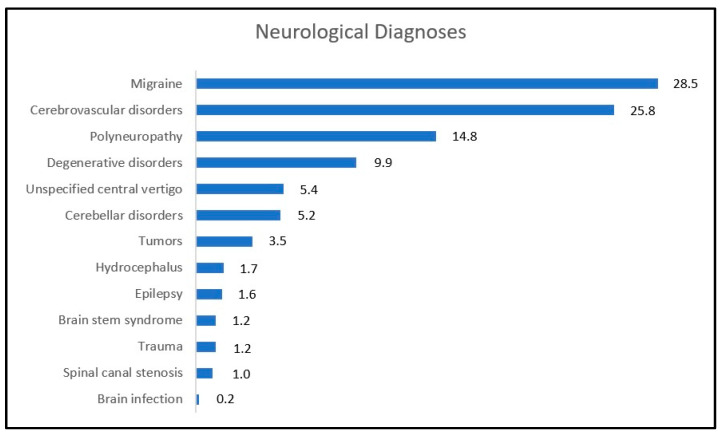
Neurological diagnoses within the selected studies. All numbers are shown in percentages.

Neurological disorders were heterogeneous involving mainly central nervous system diseases (see Figure 5). Migraine, of which 35% was a vestibular migraine, was a common condition in this cohort, accounting for 28.5%, followed by cerebrovascular disorders (25.8%) and polyneuropathy (14.8%).

Degenerative disorders (9.9%) included Parkinson, Alzheimer, and Multisystem Atrophy. Unspecified cerebellar syndromes, CANVAS (Cerebellar Ataxia, Neuropathy, Vestibular Areflexia Syndrome), and down-beating nystagmus were categorized in the Cerebellar disorder subgroup, which accounted for 5.2% of neurological diagnoses. Tumours identified in this search included vestibular schwannoma, glomus tympanicum, meningioma and ependymoma.

Psychiatric disorders were recognized to be responsible of vertigo symptoms in 9.1% of patients (Figure 2). Data on psychiatric diseases have been included in four of the candidate papers [7,8,9,11]. In particular, anxiety was the most common reported diagnosis, accounting for 61% of cases, followed by depressive episodes; in 3% of cases, the category of psychiatric diagnosis was not specified.

Finally, ophthalmologic and musculoskeletal disorders reported in 7.5% and 6.3% of the cases respectively. Adverse medication effects and metabolic related diseases were also mentioned among the causes. In 3.4% of cases, the etiology remains unclear.

## 4. Discussion

Aging is a process that involves the entire body, with the deterioration of multiple systems; the onset of an acute or a chronic condition could dramatically affect a frail equilibrium causing a general deterioration of the health condition. Considering the different diseases that may arise in the elderly, vertigo and dizziness present a prevalence above 30%, frequently associated with other systemic conditions [18]. The fact that many vertigo patients present multiple etiologies, suggests that vertigo in the elderly is a multidisciplinary issue. Our review data confirm that audiovestibular, cardiovascular and neurological condition represent the vast majority of diagnoses causing this disorder, particularly in the elderly [19].

A comprehensive evaluation of the elderly with vertigo should be always performed, in order to identify the possible underlying pathologies. In particular, in the case of an acute presentation of vertigo, the recognition of a life-threatening disease, such as a cardiovascular or a cerebral vascular accident, is crucial and eventually the emergency/urgent treatment is compulsory. Thus, the clinical evaluation should focus first on ruling-out cardiovascular and neurological conditions. Patient anamnesis is always very important for understanding the clinical scenario. Red flag symptoms, such as presyncope, need to be identified and should guide the decision-making process and the subsequent management. However, often vertigo occurs in a picture of a multisystemic geriatric syndrome, in which the aging processes involve the audiovestibular, visual, neurological, vascular, and proprioceptive systems [20,21]; this can complicate the description of the clinical experience for patients and the differential diagnosis for the examiners [22]. In any case, bed side examination is fundamental to recognise alarming symptoms, such as the presence of atypical nystagmus (i.e., gaze-evoked or spontaneous vertical nystagmus). A well-designed patient approach, as suggested by the STANDIG protocol [23], is a valuable tool in the identification of crucial signs.

BPPV is the most common AV pathology, with: (i) a higher prevalence in the elderly compared to the younger population; and (ii) higher degrees of residual dizziness [24]. Endolymphatic hydrops represents the second most prevalent AV disorder in older adults [25,26]. In addition, as observed in presbyopia and presbyacusis cases, the aging processes affect the function of the peripheral vestibular system, causing a condition known as presbivestibulopathy. According to the Baranay Society [2], this term refers to a chronic syndrome characterized by imbalance, gait disturbance, and/or recurrent falls in the presence of bilaterally reduced function of the vestibulo-ocular reflex (VOR), that usually manifests with other systemic disorders such somatosensory, visual, and auditory impairments. When peripheral (sensory) and central balance systems fail to provide adequate balance control, the patient can experience chronic disequilibrium a condition in which the risk of falls is increased. In this context, the application of the functional Head Impulse Test (fHIT), designed to assess the VOR at cortical level, can provide further information on the integration of visual and vestibular inputs. The test evaluates the ability to accurately recognize a picture that is quickly displayed during VOR activation. A recent study demonstrated that, when the multisensory integration deteriorates (i.e., fHIT scores lower than 80%), the risk of fall increases 3.9 times [27].

In the present review, the combined alteration of visual feedback and the presence of musculoskeletal disorders were identified as the main cause of vertigo in 7.5% and 6.3% of the cases respectively. These patients may benefit of tailored rehabilitative programs which include visual, vestibular, proprioceptive exercises, and training on visual–spatial working memory [28].

The inability to walk, while performing other tasks, is one of the first manifestations of cognitive decline and the fHIT may even serve as a useful tool for identification of patients in early stages. Prompt recognition of this complex clinical picture could eventually prevent a perception of declined quality of life, isolation, depression and limited self-autonomy.

A considerable percentage of subjects (9.1%), included in this review, presented a psychiatric diagnosis, such as depressive syndrome and anxiety, which can hamper the quality of life perception. In fact, these conditions can increase the fear of vertigo attacks and subsequently the fear of falling, therefore limiting daily activities and estabilishing of a vicious circle. In particular, the possible role of anxiety as a trigger of vestibular symptoms in the elderly has been highlghted in recent years [29].

Interestingly, van Vugt, Muller and van Leeuwen [7,8,11] identified another possible concurrent source of vertigo in the elderly, which is polypharmacy. In a variable percentage of elderly patients under this condition (i.e., prescription of more than three separate drugs) the occurrence of vertigo is significantly increased [30]. Antihypertensive drugs and medications with collateral/direct effects on the central nervous system, especially sedative-hypnotic, are the main involved prescriptions, commonly administrated as chronic therapy. During the medical interview a full drug anamnesis is mandatory in order to investigate and rule-out possible drug interaction or side effects.

Finally, metabolic diseases, related to an altered glucose homeostasis as observed in diabetes, but also in hypoglycemia, were described in 3.6% of cases, while in a small but not negligible, percentage of patients (122 cases or 3.4%), the etiology remains unknown.

Strengths. This paper offers updated information of the most common causes of vertigo in the elderly, identifing the three major subgroups (audiovestibular, cardiovascular and neurological). Interestingly, the possible role of psichiatric disorders has been also highlighted in this age group, by this review.

Drawbacks. Major limitations of this review is the selection of participants, since the clinical design of each study is not homogeneous (ranging from primary to tertiary care centres). Furthermore, due to the multidisciplinary nature of this disorder, the authors background could have impacted the identification of the primary underlying etiology and other possible co-factors. In addition, in this elderly population with frequently multiple comorbidities, it is difficult to determine which cause has a key role in the onset of vertigo. Unfortunately, it has not been possible to identify the secondary diagnoses and their relative frequency, since the data were inconsistent between the candidate papers.

## 5. Conclusions

Vertigo represents one of the most common problems in the elderly and also a serious condition, often leading to increased morbidity and mortality. According to the results of the present review, audio-vestibular disorders, and among these BPPV, represent the most frequent cause of vertigo; a greater awareness of these conditions should be advocated among specialists dealing with patients of this age group. Moreover, clinicians should always carefully investigate these patients, in order to identify the different factors underlying this condition and the eventual concurrent pathologies.

## Figures and Tables

**Table 1:** List of papers included in the review.

Authors	Year	Country	#	Sex	Age	Study
Müller KJ et al. [7]	2022	Germany	707	M: 344, F: 363	64–78	P
van Vugt VA et al. [8]	2020	Netherlands	417	M: 110, F: 307	65–90	P
Pan Q et al. [9]	2018	China	129	M:55, F: 74	60–90	P
Mangabeira Albernaz PL [10]	2014	Brazil	164	M: 69, F: 95	65–90	P
van Leeuwen RB et al. [11]	2014	Netherlands	731	M: 254, F: 477	>70	P
** Summary **	2014–2022	_	2148	M: 832, F: 1316 M:F = 1:1.58	60–90	_

(P = prospective study, # = number of patients).

## Data Availability

Not available.
